# Volumetric Cone Beam Computed Tomography for the Assessment of Oral Manifestations in Systemic Sclerosis: Data from an EUSTAR Cohort

**DOI:** 10.3390/jcm8101620

**Published:** 2019-10-04

**Authors:** Cristina Iordache, Magda-Ecaterina Antohe, Rodica Chirieac, Eugen Ancuța, Oana Țănculescu, Codrina Ancuța

**Affiliations:** 1 Department of Implantology, Removable Dentures, Technology, Ergonomics Discipline, School of Dental Medicine, Grigore T. Popa University of Medicine and Pharmacy of Iași, Iasi 700115, Romania; ccmiiordache@yahoo.com; 2 Department of Implantology, Removable Dentures, Technology, Removable Denture Discipline, School of Dental Medicine, Grigore T. Popa University of Medicine and Pharmacy of Iași, Iasi 700115, Romania; 3 Sancare Medical and Research Center, Iasi 700503, Romania; chiriac01ro@yahoo.com; 4 Elena Doamna Clinical Hospital, Research Department, Iasi 700398, Romania; ancuta.codrina.irena@scr.ro; 5 Department of Odontology-Periodontology, Fixed Prosthodontics, School of Dental Medicine, Grigore T. Popa University of Medicine and Pharmacy, Iasi 700115, Romania; 6 Department of Rheumatology, School of Medicine, Grigore T. Popa University of Medicine and Pharmacy, Iasi 700115, Romania; codrina.ancuta@umfiasi.ro

**Keywords:** cone beam computed tomography, systemic sclerosis, widening of periodontal ligament space, mandibular erosions

## Abstract

Background: Oral health issues are commonly reported in systemic sclerosis (SSc), comprising a broad spectrum of manifestations, e.g., reduced mouth opening, periodontal disease, increased periodontal ligament (PDL) space width, and mandibular resorption. We aimed to assess oral radiographic abnormalities, particularly PDL space widening and erosions, and to identify potential relations with disease measures. Methods: cross-sectional study in 43 SSc and matching controls receiving systematic oral assessments (full mouth dental/periodontal) and imaging (radiographs and cone beam computed tomography (CBCT)). Associations between disease variables and radiologic findings were investigated by univariate and multivariate analysis (SPSS-v.20, *p* < 0.05). Results: CBCT demonstrated generalized PDL space widening in up to half SSc, with at least one tooth involved, essentially in the posterior region (*p* < 0.05). Significant correlations between number of teeth with PDL space widening and disease severity, skin score, disease subset, topoisomerase I specificity, age, and disease duration were reported (*p* < 0.05). Additionally, mandibular erosions were described in one out of four patients, commonly condylar erosions. Conclusions: Tridimensional CBCT approach confirmed widening of PDL and mandibular erosions as common dental findings in scleroderma. Furthermore, widened PDL spaces correlated with several disease characteristics including severity, skin extent, and antibody profile.

## 1. Introduction

Systemic sclerosis (scleroderma) is a chronic multisystem disease characterized by a dynamic and exclusively complex pathobiology directed by three essential processes: autoimmunity, widespread obliterative vasculopathy of small arteries causing ischemia-reperfusion injury, and varying degrees of inflammation and tissue fibrosis [1,2]. 

The hallmark of this rare connective tissue disorder is its highly variable expression, with clinical phenotypes defined by different stages of disease expression, multiple organ involvement (lung, heart, kidney, gastrointestinal tract, and musculoskeletal system), and specific serologic biomarkers (anti-topoisomerase 1, anti-centromere, and anti-RNA polymerase III antibodies), resulting in significantly altered quality of life and survival [1,2].

Although recent guidelines tried to redesign therapeutic strategies to prevent progression and to minimize damage of specific organ involvement, the management of scleroderma remains a challenge in routine practice [1,2].

Oral health issues are commonly reported in patients with SSc (up to 80%) and comprise a broad spectrum of manifestations, from reduced mouth opening (microstomia) with abnormal interincisal distance and decreased salivary flow (xerostomia) to increased number of missing teeth and caries, periodontal diseases with gingival recession or other types of oral infections, and even temporomandibular joint involvement [1,2,3,4,5,6,7].

A number of explanations concerning the interfaces between SSc and oral health have been proposed; it seems that orofacial manifestations are generally related to excessive and extensive fibrosis of skin and oral mucosa, as well as to local vasculopathy [1,3,4,5,6,7]. Likewise, poor oral hygiene prompted not only by local conditions (microstomia) and compromised gingival vascularization but also by hand disability as a result of sclerodactyly, acro-osteolysis, and/or small joint arthritis may compromise oral health [1,2].

Since oral disease represents an additional contributor to altered quality of life [8] and dental treatment could be more difficult or, sometimes, unrealistic in advanced disease, routine oral assessment is indicated in scleroderma patients aiming at early detection and management of specific dental problems [9,10]. 

Two major radiographic features are described in scleroderma, widening of the periodontal ligament (PDL) space [11,12,13,14,15,16,17] and mandibular erosions [12,15,16,17,18,19,20]. 

PDL represents the soft tissue between the inner wall of the alveolar pocket and the roots of the teeth, consisting mainly of type I collagen and connecting the cementum of teeth to the gingivae and alveolar bone [14,15,16,17,19,21]. 

A uniform widened PDL space at least twice as normal occurs in up to two-thirds of cases and is thought to be linked to an increased collagen synthesis in the ligament as a part of global fibrosis that characterize scleroderma and is not indicative of periodontal disease in the absence of occlusal trauma [15,16,17,18,19]. 

Bone erosions are identified at the sites of muscle attachment in the mandible: the masseter at the angle of the mandible, the lateral pterygoids at the condylar head, the temporalis at the coronoid process, and the digastric at the digastric region [14,15,16,17,19].

Various papers have rated the frequency of such imaging abnormalities in SSc, mainly using two-dimensional radiographs [9,14,15,16,17,20], while the performance of Cone Beam Computed Tomography (CBCT) was assessed in few case reports [21,22,23]. 

Since three-dimensional (3-D) data reconstruction of the intraoral conditions using digital tools provide additional evidence to detect damaging manifestations within the oral cavity and is actually extensively used in the dental field for recognizing the location and severity of lesions in small anatomical regions (two or three teeth), we supposed that 3-D CBCT may also be useful to identify and, therefore, to better characterize scleroderma-related oral pathology [21,22,23].

This study aimed to assess oral radiologic manifestations associated with scleroderma using high-resolution CBCT and to identify potential relations with disease variables.

## 2. Material and Methods

We performed a cross-sectional study in a cohort of forty-three consecutive patients fulfilling either the old 1980 ACR (American College of Rheumatology) criteria or the recent 2013 ACR/EULAR (European League Against Rheumatism) classification criteria for SSc, attending at least once the outpatient rheumatology department (Clinical Rehabilitation Hospital of Iasi, Romania) during a twelve-month interval (January 2017–January 2018). Additionally, forty-three sex- and age-matching controls were recruited from patients addressing osteoarthritis at the same department.

The study was approved by the ethics committee board of Clinical Rehabilitation Hospital of Iasi, and subjects provided written informed consent before enrollment.

Patients underwent rheumatologic and routine oral health evaluation; in addition, all individuals were referred for dental radiographic evaluation. 

### 2.1. SSc-Related Parameters 

Specific scleroderma data comprised different variables as defined by the standard requirements promoted by EUSTAR (European Scleroderma Trials and Research Group) and included subset classification, meaning limited or diffuse cutaneous SSc based on their skin extent (LeRoy classification criteria); disease duration; clinical profile (modified Rodnan skin score 0–51, digital vasculopathy. and other visceral involvement); antibody profile (anti-topoisomerase-1, anti-centromere antibodies, and anti-RNA polymerase III); disease activity (revised EUSTAR activity index (0–3)); and severity (Medsger disease severity scale; 0–4) [2,24,25,26]. Limited cutaneous SSc (lcSSc) was defined as skin involvement distal to the elbows and knees, while diffuse cutaneous SSc (dcSSc) as skin involvement proximal to the elbows and knees, with or without truncal involvement [1,2].

### 2.2. Oral Manifestations

Detailed oral examinations were conducted at the Dental Learning Clinical Department of Grigore T. Popa University of Medicine and Pharmacy Iasi, Romania, and included dental and periodontal measurements according to a standardized plan, e.g., interdental distance, number of missing teeth, restored teeth, carries and periodontal indices performed by two trained dentists aiming to identify pathological tooth migration, occlusal trauma, periapical pathology, and periodontal disease.

Interdental distance was calculated between the incisal edge of the lower central incisor tooth to the incisal edge of the upper central incisor [14,15,16,27]. 

Plaque accumulation reflecting oral hygiene status was recorded with a Silness and Loe plaque index (PI), while gingival index (GI) was used to measure the extent of inflammatory gingivitis [28]. Both indexes were evaluated as degrees from 0 to 3 [29] and also the percentage of sites with detectable plaque (Pl%) and with bleeding on probing (BOP%) [30].

Periodontal status was appreciated by periodontal probing depth (PPD), the distance from the gingival margin to the base of the gingival sulcus, and by clinical attachment level (CAL), the distance on the buccal or labial surface from the cementoenamel junction to the base of the gingival sulcus [14,15,16,17,31,32]. We used a custom periodontal chart that included the measurement of the gingival recession and the pocket depth in 4 points (mesiobuccal, distobuccal, mesiolingual, and distolingual) for each tooth, assessed with a periodontal probe marked in millimeter intervals [21]. The presence of periodontal disease in a given tooth was defined as either a PPD > 3 mm or a CAL ≥ 5.5 [14,15,16,17,30,31]. The extent of periodontal disease in the entire mouth was calculated as the number of involved teeth [14,15,16,17], and the severity (no, mild, moderate, or severe periodontitis) was also assigned accordingly [14,15,16,17]. 

Temporomandibular joint (TMJ) involvement (pain, sounds, and mobility) was also recorded. 

### 2.3. Imaging Studies

All patients had a detailed radiologic analysis meaning conventional radiographs and an upper and lower jaw volumetric CBCT, focusing on the width of the periodontal ligament space and the presence of mandibular erosions. 

Radiographically, PDL occurs as the radiolucent space between the lamina dura and the tooth root; its normal width ranges between 0.15 mm and 0.21 mm and decreases with age [21]. The presence of a widened PDL was recorded on both panoramic radiographs and CBCT and appreciated as widening near the coronal portion of the root or in the periapical region in either one of both sides of the root [14,15,16,17].

Orthopantomogram (OPG) offers a preliminary assessment of both teeth and temporomandibular joint and were preferred; however, bitewings (used for the detection of caries, inflammatory pulp changes, and proximal periodontal irritant factors) and periapical radiographs (for only 2 to 3 teeth) were also performed in selected cases (protocol adapted from Canadian Oral Health Study) [14,15,16,17]. 

Since 3-D analysis of the oral cavity facilitates the evaluation and diagnosis of different intraoral conditions including TMJ and different periodontal issues, 3-D CBCT was systematically done in all subjects. CBCT provides additional data for the accurate assessment of the buccal and oral alveolar bone of which the resorption is not visible on panoramic images and is also recommended for the visualization of mandibular condyle as well as for the complete evaluation of the periodontal space emphasizing areas with maximal alveolar bone loss.

The CBCT scanner used was *Planmeca Promax 3D Mid (Planmeca OY, Helsinki, Finland)*. Images were obtained at 90 kV and 12 mA with a 0.2 voxel size, and the typical exposure was 5.4 s. NNT® NewTom software (Quantitative Radiology, Verona, Italy) was used to evaluate the CBCT images. Sagittal and coronal (1-mm thickness) as well as axial sections (0.5-mm thickness) of the condyle were evaluated. Readings was performed by the same trained oral radiologist.

PaX-i3D Green CBCT (Vatech, Hwaseong, Korea) and Ez3D Plus Professional CBCT software (Vatech Co., Hwaseong, Korea) (version 1.2.6.23) were used as 3D viewer and image analysis software. Images were obtained at 94 kV, 7 mAs, 0.120 voxel size, 9-s exposure time, 1–0.5-mm slice interval, and 0.5–0.2-mm slice thickness. 

The CBCT measurements were done on the teeth without clinical signs of active periodontitis, occlusal trauma, or pulpal injuries.

In the first step, it was checked if CAL < 5 mm and the lamina dura is present. The measurements were initially made on the coronal and sagittal sections, from the cementoenamel junction to the alveolar crest. If CAL > 5 mm, the tooth was included in “periodontal group”. Subsequently, on 3-D reconstruction, the areas with resorption potentially greater than 5 mm were identified. The measurements were made at those levels, completing the measurements in axial and sagittal plane, and if they were really larger than 5 mm, the tooth was included in “periodontal group”.

In the next stage, the periodontal space measurement was performed on axial sections in the cervical, middle, and apical third of the clinical root, considering the perpendicular between the root surface and the lamina dura. In the cervical third, the PDL space was evaluated in 8 points: in the middle of each root face (buccal—B, oral—O, mesial—M, and distal—D) and at the transition angles (MB, DB, DO, and MO). In the middle and apical third of the clinical root, the measurements were made on the middle of each root surfaces. Additionally, the evaluations were completed with an apical measurement on the sagittal or axial section from the tip of the root to the lamina dura in the cervico-apical direction (apical periodontal space).

For the multirooted teeth, measurements were added at the furcation level on the sagittal or axial section in the cervico-apical direction.

For each region, the average was determined.

We were interested only in teeth with regularly widened PDL without any marginal or apical periodontal lesion; for a cervically enlarged PDL, we searched for potential putative factors (e.g., occlusion trauma, orthodontic tooth movement, irritative mechanical, or chemical factors) and excluded it from the final analysis. Occlusal trauma was clinically identified by abrasion facets, premature contact points, or interferences in static and dynamic occlusion. 

Patients with a history of orthodontic treatment were not enrolled in the study.

Mandibular erosions (condyles, coronoid processes, digastric regions, and posterior rami) were also recorded using panoramic radiographs and CBCT sections as well and rated by using a Likert-type scale from 1 to 5, from “definitely no erosion, 0” to “definitely erosion, 5”, that was adapted from [14,15,16,17].

### 2.4. Statistical Analysis

Descriptive statistics were used for scleroderma parameters and radiologic manifestations of the patients with SSc and the controls. Nonparametric statistics (Spearman rank correlation; univariate and multivariate analysis adjusted for age, gender, and smoking status) were used to analyze the association between SSc and number of teeth with widening of the PLS and erosions. Mann Whitney U tests, Chi-squared tests, and Fisher’s exact tests were used accordingly.

All statistical analyses were performed using SPSS (Statistical Package for Social Sciences) v.20 IBM statistics (IBM Corp., Armonk, NY, USA) with *p*-values < 0.05 being considered statistically significant.

## 3. Results

### 3.1. SSc-Related Characteristics

We enrolled 43 subjects with scleroderma as follows: mainly women (72%, *n* = 31), with a mean (SD) age of 43.95 (11.36) years and a mean (SD) disease duration of 8.7 (4.5) years; 67.74% (*n* = 29) had diffuse disease, and 53.48 % (*n* = 23) were anti-topoisomerase 1 positive SSc; and the mean (SD) disease severity was 4.8 (2.1) (Table 1).

The same number, sex, and gender of matching controls were recruited.

### 3.2. Oral Health

We demonstrated a reduced maximal mouth opening in 69.76% patients (*n* = 30) and a mean (SD) inter-incisal distance of 32.5 (7.2) mm, smaller than normal (*p* < 0.05).

The mean (SD) number of evaluable teeth was 23.5 (4.2) in SSc and 29.6 (2.1) in controls, respectively, with a trend to have more missing teeth in patients with SSc; moreover, these individuals were significantly more likely to be edentulous than matching controls (Table 1). 

At *n* = 27, 62.8% of SSc experienced one or more caries, and more than half of patients (53.48%, *n* = 23) presented with periodontal disease.

Significantly higher plaque accumulation was found in SSc, up to 50% of patients displaying sites with detectable plaque: 0.75 (0.39–1.51) vs. 0.39 (0.24–0.61) in controls (*p* < 0.05). Furthermore, gingival inflammation was found in 67.44% (*n* = 29) cases, while 55.81% of scleroderma patients (*n* = 24) presented with bleeding on probing. 

For *n* = 14, 32.55% SSc had periodontal pockets and 27.9% (*n* = 12) had a CAL ≥ 5.5 mm; mean (SD) PD was significantly different in scleroderma compared with controls: 5.21 (0.25) mm vs. 3.15 (0.37) mm, *p* < 0.05. Severity of periodontitis was also meaningfully different in SSc vs. controls (*p* < 0.05), with severe disease described in up to one third of scleroderma and related periodontal disease (Table 2).

Likewise, considerably more SSc presented clinical symptomatic TMJ involvement (*n* = 18) as compared with controls (*n* = 7) (*p* < 0.05). 

### 3.3. Imaging Studies

Panoramic radiographs were performed in all cases to allow a basic assessment of the mandibular erosions and to select teeth suitable for further CBCT evaluation. Both panoramic radiographs and CBCT sections showed the widening of the PDL space, several remaining roots, and dental caries; in addition, mandibular erosions, even condylar lysis, were described, particularly using CBCT exams.

#### 3.3.1. PDL Space Widening

Panoramically reconstructed CBCT demonstrated widening of the PDL in at least one tooth in 46.51% SSc (*n* = 20) vs. 13.95% (*n* = 6) controls (*p* < 0.05). Mean (SD) periapical PDL width was 0.35 (0.16) mm, about twice the normal thickness, and 0.17 (0.04) mm in controls (*p* < 0.05) 

Although both anterior and posterior teeth were involved, a wider PDL was commonly found in the posterior region (*p* < 0.05). Nevertheless, the molars and premolars presented with the most significant differences between scleroderma and controls (*p* < 0.05).

Multivariate analysis adjusting for age, gender, and current smoking status confirmed the important difference between the two groups (OR 7.26; 95% CI 3.87–13.65). Moreover, there were significantly more teeth with widened PDL in scleroderma patients than in controls as suggested by univariate and multivariate statistics: 1.21 (2.31) vs. 0.28 (0.25) (*p* < 0.05), with relative risk (RR) 8.51; 95% CI 5.37–12.61), respectively. 

No calcification within the PDL space was demonstrated.

**Figure 1:**
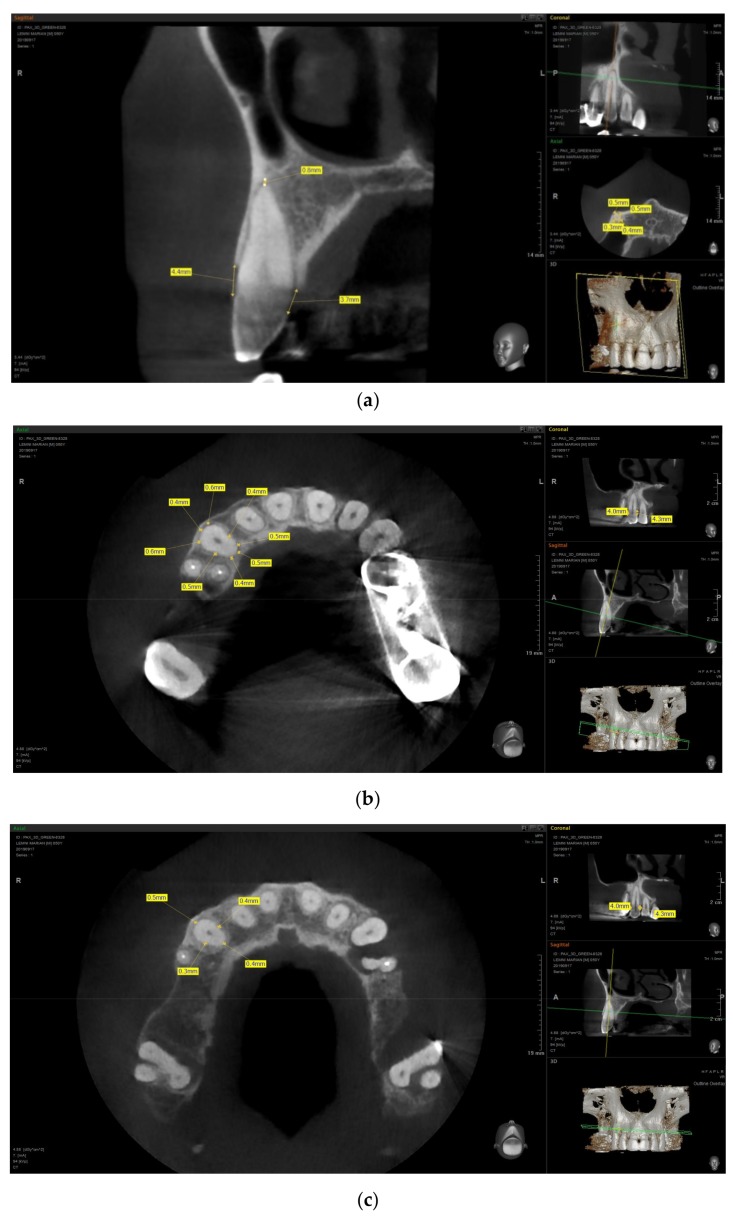

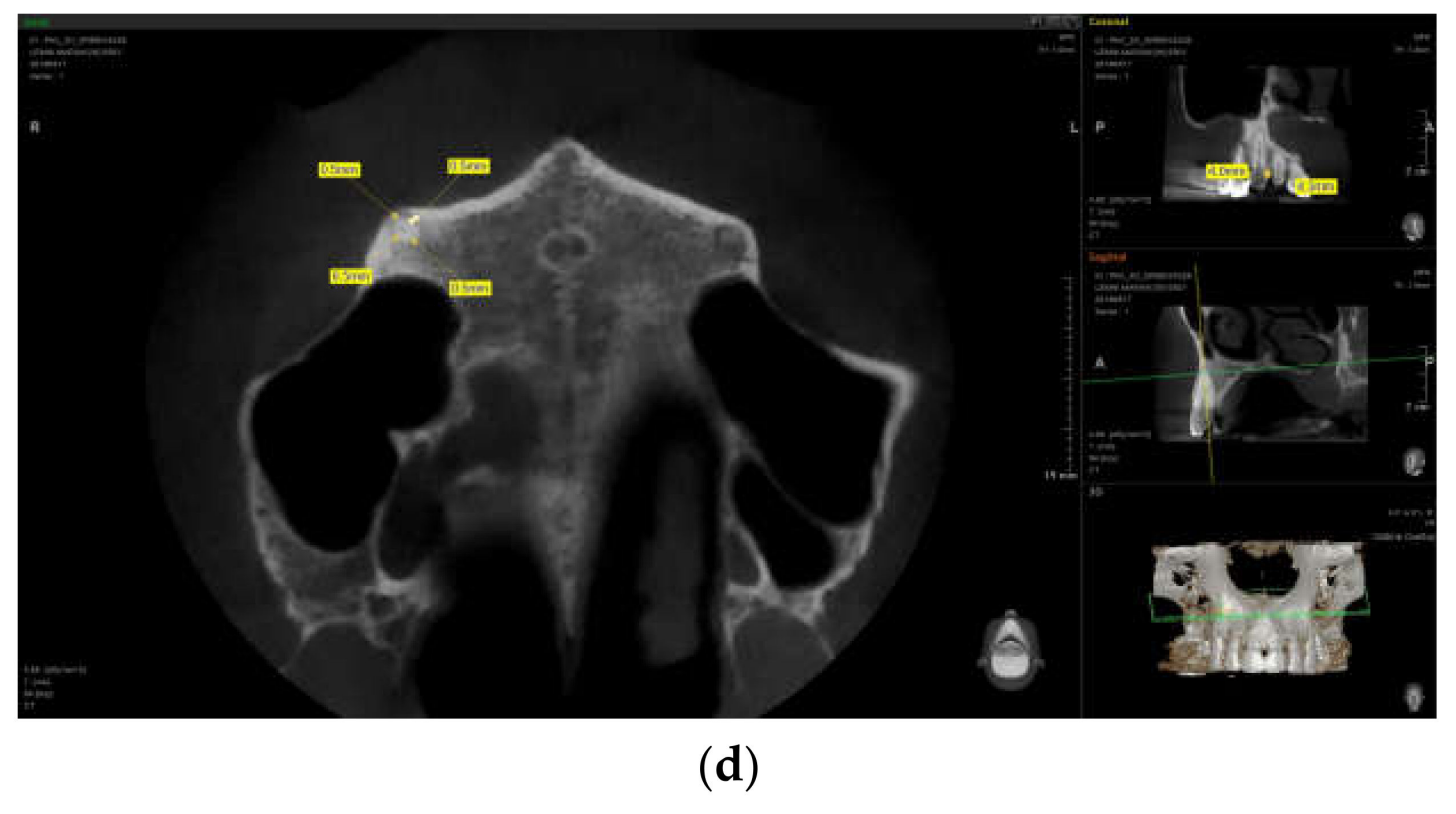
Measurements of periodontal ligament space on cone beam computed tomography (CBCT): (**a**) clinical attachment level (CAL) and apical PDL space measurements; (**b**) 8-point cervical third measurements; (**c**) 4-point middle third measurements; and (**d**) 4-point apical third measurements.

Figure 1 presents examples of PDL space measurements. 

#### 3.3.2. Erosions

**Figure 2:**
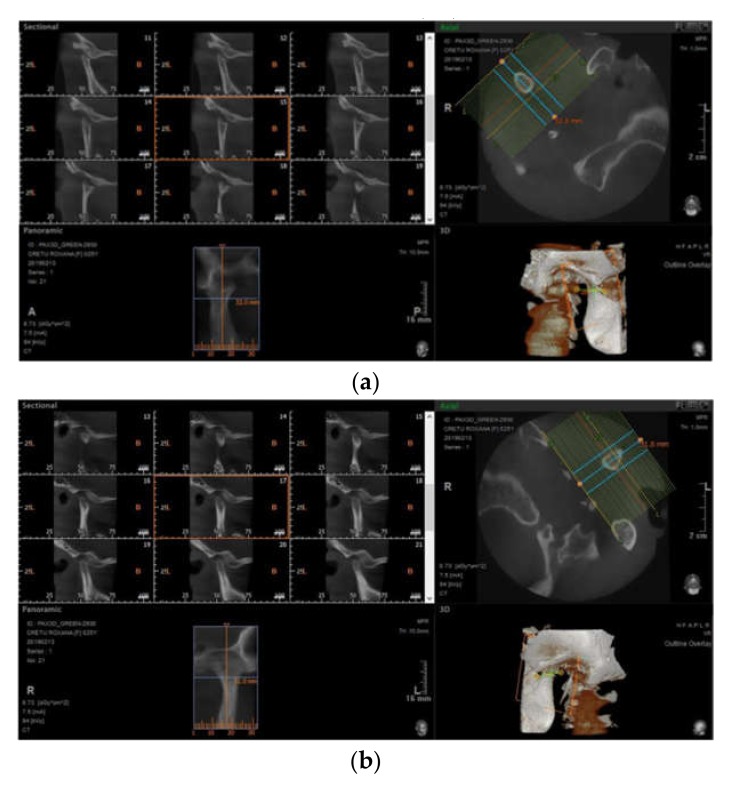
CBCT aspects of the (**a**) right and (**b**) left temporomandibular joint (TMJ).

**Figure 3:**
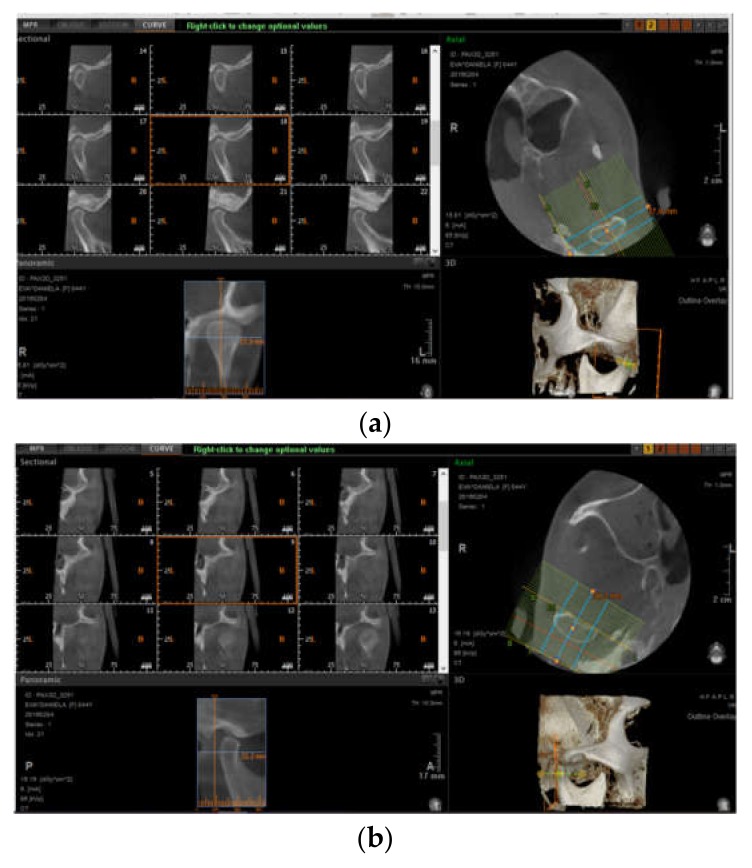
CBCT aspects of the (**a**) right and (**b**) left TMJ.

Additionally, erosions of the mandible were described in 23.25% (*n* = 10) of scleroderma cases (Table 1, Figure 2 and Figure 3), with the majority of patients presenting with at least one condylar erosion; the mean (SD) number of sites with bone erosions was 0.42 (0.5).

Overall, patients with SSc had more mandibular erosions, irrespective of the locations (condyle, coronoid process, or otherwise) vs. controls (23.25%, *n* = 10 vs. 6.97%, *n* = 3, *p* < 0.05) as demonstrated by multivariate analysis (OR 5.32; 95% CI 1.87–11.74).

However, the difference was significant only for the condylar erosions in patients with SSc compared with control (18.60%, *n* = 8 vs. 232%, *n* = 1; *p* < 0.05). It may be only because the changes in the condyles are easier to detect compared to the others, especially in the early stages. In addition, changes due to stomatognathic system dysfunction (malocclusion, edentulism, maxillo-mandibular malrelationship) may also be superimposed.

### 3.4. Associations with PDL Space Widening and Erosions

Subgroup analysis in scleroderma subjects with and without PDL widening and with and without erosions based on univariate analysis including clinical parameters and oral radiographic abnormalities were, further, done (Table 2). 

Significant correlations between number of teeth with PDL widening and MEDSGER disease severity score (*r* = 0.702, *p* = 0.028), RODNAN skin score (*r* = 0.821, *p* = 0.01), disease subset (diffuse vs. limited, *r* = 0.782, *p* = 0.041 vs. *r* = 0.451, *p* = 0.05, respectively), anti-topoisomerase I antibodies (*r* = 0.568, *p* = 0.012), age (*r* = 0.872, *p* = 0.01), and disease duration (*r* = 0.811, *p* = 0.01) were demonstrated.

Moreover, the relative risk of PDL space widening in relation with different determinants of scleroderma severity are listed in Table 3.

For multivariate analysis adjusting for age, disease duration, gender, and smoking status, there were a significant association between the number of teeth with widened PLD and MEDSGER disease severity (*p* < 0.05) (Table 4).

**Figure 4:**
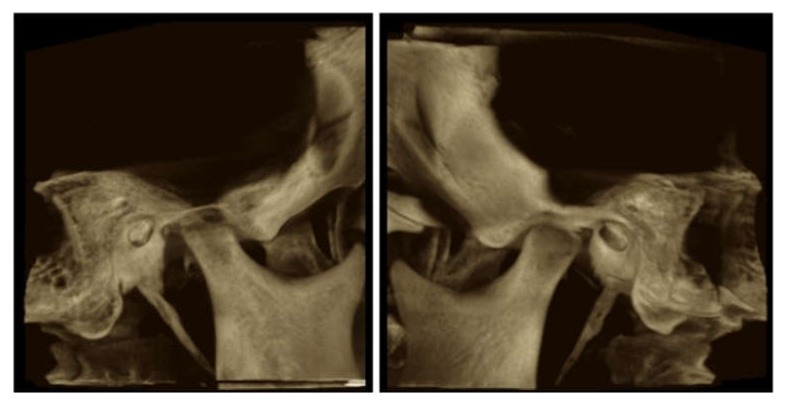
CBCT-condylar lysis and condylar remodeling.

Erosions were predominantly found in patients with aggressive scleroderma, especially related to the attachment of lateral pterygoids at the condylar head and masseter muscle at the angle of mandibula. Total resorption of the condylar head was reported only in one case (Figure 4).

Also, there was an inverse association between interdental distance and the number of erosions (RR 3.51, 95% CI 1.67–2.95, *p* < 0.05) (Table 2). 

## 4. Discussion

We performed a systematic 3-D analysis of the radiographic orofacial abnormalities associated with SSc, aiming to describe specific CBCT findings and to identify potential correlations with disease characteristics, activity/severity, or prognostic factors. This was a case–control study within an EUSTAR SSc-cohort and matching controls focused on a comprehensive evaluation of periodontal ligament and erosions as the most common radiographic manifestations in scleroderma. 

We reported an increased rate of PDL space widening with a tendency for significantly more teeth with widened PDL space in scleroderma as compared to controls irrespective of the evaluation method (number of teeth with abnormal PDL or individual evaluation per one tooth): 46.51% SSc with PDL widening in at least one tooth vs. 13.95% controls. Moreover, wider PDL space was generally detected in the posterior teeth in more than one quadrant, although anterior region was also involved. Our results are within the range of reported data in literature by using conventional panoramic radiographs (38–66%) [9,14,15,16,17,20,32,33,34].

A systematic review of the literature addressing the impact of scleroderma on oral health has identified only a few studies having as endpoint the radiographic findings; moreover, the majority considered bi-dimensional panoramic radiographic assessment, and none of them focused on interrelations between imaging parameters and disease characteristics [9,14,15,16,17,20,32,33,34]. 

A closer look to the Canadian Systemic Sclerosis Oral Health Study offered a relevant picture of oral manifestations related to SSc; this was an ambitious study focused on SSc interferences with oral conditions within a large cohort of patients selected from several rheumatology centers. Compared with controls, patients with SSc are significantly more likely to have more teeth (molars and premolars) with PDL space widening as well as mandibular erosions. Furthermore, the evaluation of radiographs by two blinded experts increased accuracy of the results [14,15,16,17].

We performed a panoramic approach of oral SS-related features by two-dimensional radiographs and also by high-resolution volumetric CBCT. Although the oral pattern of involvement in scleroderma on standard films is widely recognized, data on 3-D assessments are scare. In fact, only one study with a comparable design is still ongoing in a French cohort aiming to characterize precisely the oral manifestations associated with SSc and to identify specific radiological (3-D CBCT), clinical, and/or biological signs [35]. 

We used Adobe Photoshop for PDL space widening assessment on panoramic images; compared to the Canadian SSc Oral Health Study, where results were analyzed by two radiologists, in our study, data were read by only one radiologist, and this may have a potential bias for the accuracy of the results [14,15,16,17]. 

PDL remains an active tissue, with an increased remodeling capacity [15,16,17]. Widening of the PDL space may be related to SSc as a result of excessive and extensive fibrosis of the ligament correlating with disease severity [1,7] and also may develop in occlusal/orthodontic trauma, periodontal/periapical disease, pulpo-periapical lesions, bisphosphonate-related jaw osteonecrosis, malignancies (osteosarcoma, chondrosarcoma, and non-Hodgkin lymphoma) or in radiation-induced bone defect [19]. In dental occlusion trauma, PDL involvement is usually related to excessive or para-axial biting forces and is generalized or located and associated with angular bone defects and teeth mobility, while in malignancies, PDL space widening is limited to the adjacent teeth and the lamina dura is involved. In contrast, in patients with scleroderma, the lamina dura frequently remains intact and a uniform enlarged PDL space occurs in more than one quadrant, usually in the posterior teeth [11,12,13,15,16,17,18,19].

On the other hand, we reported an increased frequency of mandibular erosions in about one out of four SSc (23.25%), particularly condylar erosions developed in the area of the lateral pterygoids muscle attachment to the bone (see figure). Our results are within the range reported from previous 2-D studies (7–10%); in fact, only one paper described a higher prevalence of mandibular angle involvement (83.33% cases) [14,15,16,17,36,37].

However, the difference was significant only for the condylar erosions in patients with SSc compared with controls (*p* < 0.05).

It is well established that mandibular findings in scleroderma include resorption of the angle, condyle, coronoid process, ascending ramus, and antegonial notch. However, complete condylar osteolysis and secondary mandibular resorption are only rarely documented [14,15,16,17,34]. Abnormal muscle contraction with subsequent abnormal pressure on the bone via atrophic muscles at their attachment site [15,16,17,33], bone ischemia due to vasculitis and deposition of collagen in vascular wall, as well as rigidity and pressure from overlying skin are among the mechanisms likely involved in the emerge and persistence of bone erosions in SSc [14,15,16,17,34]. 

Calcifications within the periodontal ligament were not found in our study. An interesting paper published several years ago reported calcifications within the PDL space of most maxillary teeth seen on 3-D CBCT analysis as well as pulp calcifications in some incisors and premolars with subsequent root canal obliterations [38].

Finally, we investigated associations between SSc-related variables and typical oral radiographic features in scleroderma. To our knowledge, only the Canadian Oral Health Study has systematically assessed and reported such correlations; our analysis supports data already published, considerably increasing the expertise in oral health in different scleroderma settings. Interestingly, widening of the PDL space is related to disease severity, reflecting extensive fibrosis and aberrant collagen turnover, and no association with periodontal disease measures and missing teeth were described. However, we did not observe any correlation between PDL space widening and smoking as previously described, although the influence of nicotine on fibroblast phenotype and activity within PDL space is recognized [14,15,16,17]. 

We described a significant negative correlation of mandibular erosions and interincisal distance, suggesting that abnormal local intraoral stress may be responsible for bone lesions; in addition, there was no relation with disease activity and severity. Condylar erosions were associated with clinical signs and symptoms of TMJ involvement, but this was not valuable for erosions with other location. 

Our study has some limitations, e.g., the number of recruited patients, the evaluation of radiographs by only one expert, and associated multiple oral pathologies. 

The quality of the images obtained is of paramount importance. There is a greater probability of correctly identifying and measuring fine structures like lamina dura and PDL when the image is acquired using the smallest voxel resolution parameters. On the other hand, a compromise has to be made in order to reduce patient exposure to X-rays by a shorter scanning time, using protocols that offer the same performances. In our study, the 0.4 mm voxel size was used for TMJ images, allowing a qualitative analysis, and 0.12 mm voxel size was used for PDL space measurements, allowing a quantitative evaluation.

## 5. Conclusions

To conclude, volumetric CBCT performance in assessing PDL space widening and mandibular erosions in patients with SSc shows promising results. Furthermore, PDL space widening as well as mandibular erosions are commonly reported in scleroderma and correlated with different disease characteristics especially severity, skin extent, and antibody profile. Future studies could investigate the pattern of PDL widening at different root heights and in different oral disease context. 

## Figures and Tables

**Table 1:** Systemic sclerosis (SSc)-related parameters, clinical oral, and radiographic features.

Parameter	No (%) or Mean ± SD
Demographics	
Female	31 (72)
Age	43.95 (11.36)
Smoking status	10 (23.25)
SSc-related measures	
Diffuse cutaneous SSc	29 (67.74)
Disease duration	8.7 (4.5)
Modified RODNAN (0–51)	18 (10.1)
Facial skin score (0–3)	1.5 (0.7)
Serology	
Anti-SCL70	23 (53.48)
Anti-centromere B	10 (23.25)
Dental issues	
Missing teeth per subject	9.3 (4.5)
Teeth with periodontal disease	7.2 (3.4)
PDL space widening	
Patients with uniform PDL space widening	20 (46.51)
Apical PDL space widening (mm)	0.35 (0.16)
Erosions	
Patients with mandibular erosions	10 (23.25)
Number of subjects with condylar erosions	8 (18.60)

PDL = periodontal ligament.

**Table 2:** Associations between scleroderma-related parameters and oral radiographic abnormalities (univariate analysis).

Parameter	PDL Widening		Erosions	
	RR	*p*	RR	*p*
Demographics	1.1	*p* > 0.05	1.2	*p* > 0.05
Female	1.43	*p* > 0.05	1.1	*p* > 0.05
Age				
Smoking status	1.06	*p* > 0.05	1.06	*p* > 0.05
SSc-related measures				
Diffuse cutaneous SSc	1.25	*p* > 0.05	1.02	*p* > 0.05
Disease duration	2.36 *	*p* < 0.05	0.98	*p* > 0.05
Modified Rodnan (0–51)	3.12 *	*p* < 0.05	1.3	*p* > 0.05
Facial skin score (0–3)	2.71 *	*p* < 0.05	1.02	*p* > 0.05
SSc activity	1.21	*p* > 0.05	1.17	*p* > 0.05
SSc severity	3.09 *	*p* < 0.05	0.92	*p* > 0.05
Interdental distance	1.21	*p* > 0.05	3.51 *	*p* < 0.05
Dental issues				
Missing teeth per subject	1.02	*p* > 0.05	0.87	*p* > 0.05
Teeth with periodontal disease	1.15	*p* > 0.05	1.12	*p* > 0.05

RR, relative risk; PDL, periodontal ligament; * *p* < 0.05.

**Table 3:** Associations between the number of teeth with PDL space widening and SSc severity.

Parameter	RR	95% CI
Gender	2.17	0.91–14.28
Age	1.00 *	0.89–1.73
Smoking status	5.31	4.27–9.12
SSc duration SSc subtype	3.26 * 4.51 *	1.20–7.53 2.39–8.14
RODNAN skin score SSc severity SSc activity Anti–topoisomerase 1	0.93 * 1.25 * 2.36 5.22 *	0.89–3.76 1.58–3.89 1.02–3.41 2.3–7.67

RR, relative risk; 95% CI. 595% confidence interval; * *p* < 0.05.

**Table 4:** Correlations between the number of teeth with PDL space widening and number of teeth with periodontal disease.

Parameter	RR	95% CI
Gender	2.17	0.91–14.28
Age	1.00	0.89–1.73
Smoking status	5.31	4.27–9.12
SSc duration SSc subtype	3.26 * 4.51*	1.20–7.53 2.39–8.14
RODNAN skin score SSc severity SSc activity Anti-topoisomerase 1 Number of teeth with periodontal disease	0.93 * 1.25 * 2.32 5.19 * 1.19	0.89–3.76 1.58–3.89 1.45–3.22 2.24–7.32 0.87–1.45

* *p* < 0.05.
